# Equine Pituitary Pars Intermedia Dysfunction

**DOI:** 10.3390/vetsci12080780

**Published:** 2025-08-20

**Authors:** Nicola J. Menzies-Gow

**Affiliations:** Department of Clinical Science and Services, Royal Veterinary College, Hawkshead Lane, North Mymms, Hertfordshire AL9 7TA, UK; nmenziesgow@rvc.ac.uk

**Keywords:** pituitary, pars intermedia, dysfunction, pergolide, insulin, ACTH

## Abstract

Pituitary pars intermedia dysfunction (PPID) is a common, slowly progressive, neurodegenerative disorder of the older horse. Oxidative damage to the hypothalamic periventricular neurons results in loss of dopaminergic inhibition of the pars intermedia region of the pituitary gland. Consequently, there is increased production of the pro-opiomelanocortin (POMC)-derived hormones normally produced by this region, as well as initial melanocyte hypertrophy and hyperplasia, followed by adenomatous change. Clinical signs that are highly suggestive of the disease are generalised and regional hypertrichosis and delayed/abnormal coat shedding. Numerous clinical signs provide a moderate clinical suspicion, including hyperhidrosis, abnormal fat distribution/regional adiposity, epaxial muscle atrophy/loss of topline, laminitis, weight loss, recurrent infections, behavioural changes/lethargy, polyuria and polydipsia, a pot-bellied appearance, bulging supraorbital fat pads, reduced wound healing, lordosis and infertility. In all animals, a diagnosis of PPID is made based on the signalment, clinical signs and results of further diagnostic tests, with age being a crucial factor to consider. Currently recommended further diagnostic tests are measurement of basal adrenocorticotrophic hormone (ACTH) concentrations (all year) and evaluation of the ACTH response to thyrotrophin-releasing hormone (TRH) using seasonally adjusted references intervals (non-autumn). Animals should also be tested for insulin dysregulation, as laminitis risk in PPID is associated with hyperinsulinaemia. PPID can be managed but not cured; it is a lifelong condition. The individual clinical signs can be managed, e.g., clipping the excessive haircoat and providing unrestricted access to water for individuals with polydipsia. Alternatively, pharmacological management can be employed, and the dopamine-2 receptor agonist pergolide is licensed/approved for the treatment of equine PPID. This should be prescribed in combination with dietary recommendations based on the body condition score and insulin sensitivity status of the individual animal.

## Simple Summary

Pituitary pars intermedia dysfunction (PPID) is a common disease in older equines that is associated with loss of the dopaminergic inhibitory input from the hypothalamus to the pars intermedia region of the pituitary gland. This results in overproduction of the normal pars intermedia-derived hormones. A variety of clinical signs are associated with PPID, and presence of regional or generalised hypertrichosis and/or delayed/abnormal haircoat shedding provides a high index of clinical suspicion. Numerous other clinical signs are associated with a moderate or low index of clinical suspicion. A diagnosis is based on the history, signalment, clinical signs and measurement of basal adrenocorticotrophic hormone (ACTH) concentrations and/or assessment of the ACTH response to a thyrotropin-releasing hormone (TRH) stimulation test. Insulin sensitivity should also be assessed, as laminitis occurs in the subset of animals with PPID that have insulin dysregulation (ID). PPID is a lifelong condition that can be managed using the dopamine 2 (D2) receptor agonist pergolide in combination with dietary recommendations based on the body condition score and insulin sensitivity of an individual animal.

## 1. Introduction

This review provides an overview of the pathophysiology, epidemiology, clinical signs, diagnosis and management of equine pituitary pars intermedia dysfunction (PPID). PPID is a frequently encountered neurodegenerative disorder affecting the hypothalamic dopaminergic neurones [1]. The disease appears spontaneously, is progressive and is poorly understood [2]. Oxidative damage to the hypothalamic periventricular neurons results in loss of dopaminergic inhibition of the pars intermedia region of the pituitary gland [3] resulting in overproduction of the normal pars intermedia-derived hormones [4,5]. A variety of clinical signs are associated with PPID, and presence of regional or generalised hypertrichosis and/or delayed/abnormal haircoat shedding provides a high index of clinical suspicion [6]. Numerous other clinical signs are associated with a moderate or low index of clinical suspicion [6]. A diagnosis is based on the history, signalment, clinical signs and measurement of basal adrenocorticotrophic hormone (ACTH) concentrations and/or assessment of the ACTH response to a thyrotropin-releasing hormone (TRH) stimulation test [7]. Insulin sensitivity should also be assessed, as laminitis occurs in the subset of animals with PPID that have insulin dysregulation (ID) [8,9,10]. PPID is a lifelong condition that can be managed using the dopamine 2 (D2) receptor agonist pergolide in combination with dietary recommendations based on the body condition score and insulin sensitivity of an individual animal [7].

## 2. Pituitary Gland Anatomy and Pathophysiology of PPID

**Figure 1:**
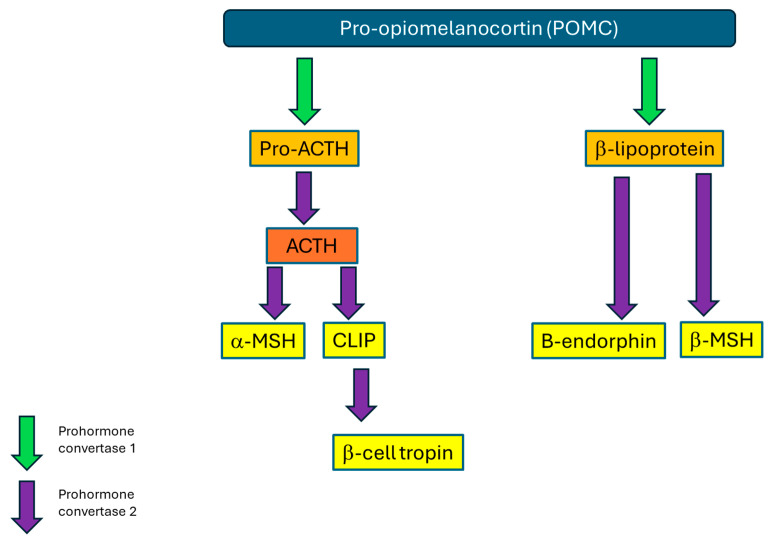
Schematic outlining the normal processing of the hormone pro-opiomelanocortin (POMC) by melanotropes within the pituitary pars intermedia.

The equine pituitary gland lies in the sella turcica at the base of the brain. It is connected to the hypothalamus by the infundibular stalk and is divided into two distinct parts, the adenohypophysis and the neurohypophysis, which have different embryological origins reflecting a difference in function. The former is further subdivided into the pars intermedia, the pars distalis and the pars tuberalis, whilst the latter is composed solely of the pars nervosa. The pars intermedia (PI) is comprised of endocrine cells known as melanotropes, which process the hormone pro-opiomelanocortin (POMC) using the enzyme prohormone convertase 1, resulting in the production of β-lipotropin and adrenocorticotrophic hormone (ACTH). Prohormone convertase 2 activity on these two products results in production of the hormones β-endorphin, α-melanocyte-stimulating hormone (α-MSH), corticotropin-like intermediate lobe peptide (CLIP) and b-cell tropin (Figure 1) [5].

The pars intermedia melanotropes are directly innervated by hypothalamic periventricular neurons, which release dopamine, resulting in inhibition of hormone production [11]. These neurones originate in the periventricular nucleus of the hypothalamus, adjacent to the third ventricle, and project through the infundibulum, terminating in the pars intermedia; dopamine acting on the dopamine 2 (D2) receptors inhibits melanotrope cell proliferation, transcription of POMC and, hence, POMC-derived hormone production [12,13]. Hypothalamic and pituitary secretions are also physiologically influenced by a range of physiologic factors, including age [14], stress [15], exercise [16], illness, photoperiod length [14,17] and climate [18], as well as illness [15,19].

**Figure 2:**
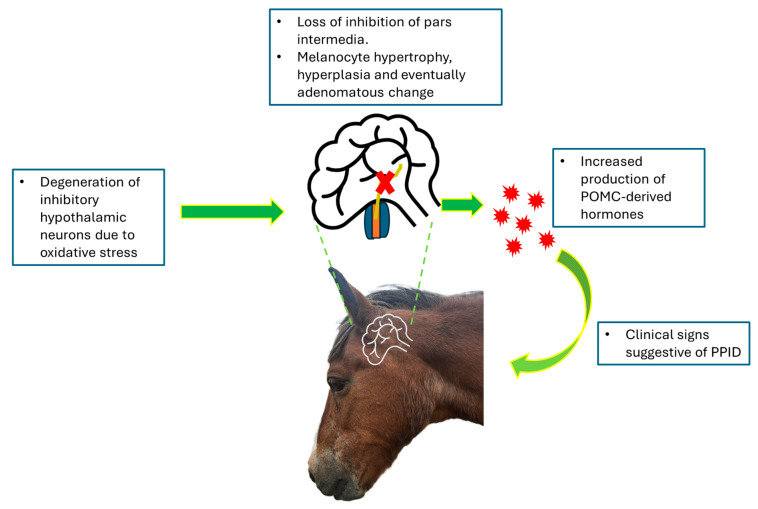
Schematic outlining the pathogenesis of equine pituitary pars intermedia dysfunction.

Post mortem studies reveal that PPID is associated with loss of the periventricular dopaminergic neurones [20] and enlargement of the pars intermedia, with initial melanocyte hypertrophy and hyperplasia followed by adenomatous change [3]. When the hypothalamic neurones degenerate, less dopamine is secreted, thus reducing the melanotrope inhibition, leading to increased PI activity; this, in turn, results in overproduction of the POMC-derived peptides normally produced by the PI (Figure 2) [4,5].

Whilst it is possible that the PI hypertrophy and hyperplasia that are features of PPID could be consequences of dopamine D2 receptor dysfunction rather than a lack of dopaminergic inhibition, a histological and immunohistochemical study revealed that, whilst horses with higher PPID histological grades had reduced tyrosine hydroxylase immunoreactivity (a marker of dopaminergic neurones), D2 receptor immunoreactivity was actually increased [2].

It should be remembered that the majority of the ACTH secreted by the pituitary comes from the pars distalis, and the ACTH secreted by the PI of horses with PPID appears to be largely biologically inactive [21]. Thus, the small increase in plasma ACTH production does not result in adrenal stimulation and consequent hypercortisolaemia [22,23]; hence, the disease is correctly known as equine pituitary pars intermedia dysfunction rather than the historical term, equine Cushing’s disease.

Oxidative stress is one potential cause of the degeneration of the dopaminergic neurones that is a feature of PPID. Dopaminergic neurones normally produce reactive oxygen species during neurotransmitter metabolism, making them particularly vulnerable to oxidative damage [20]. Oxidative stress occurs when free radicals are created as a consequence of aerobic metabolism, resulting in exposure of the cellular components to these damaging free radicals; this happens in all living organisms over time [20]. Studies have shown that staining for the marker of oxidative stress 3-nitrotyrosine (3-NT) in the periventricular neurone nerve terminals from horses with PPID was more than two-fold greater compared to aged animals without PPID, but there was no evidence of oxidative stress in the cell bodies of these neurones [20]. In Parkinson’s disease in humans, a neurodegenerative disease that has been compared to PPID, mutations in α-synuclein, a protein integral to nerve terminal function, occur [24]. Studies have revealed that there was more α-synuclein in the PI from horses with PPID compared to non-PPID horses and the 3-NT and the α-synuclein were colocalised, suggesting a link between the two [20]. Finally, in the same study, aged horses that had normal dopaminergic neurones, but evidence of oxidative stress (increased 3-NT) and increased α-synuclein expression in their PI were identified, suggesting that both these changes precede the neurodegeneration and, therefore, the development of PPID [20]. Further studies have shown that the α-synuclein extracted from the PI of horses with PPID is misfolded [25], although longitudinal studies would be needed to confirm that this misfolded protein is a cause rather than consequence of disease. The exact cause of or the initiating factor for the oxidative stress in PPID has yet to be determined, limiting understanding of the disease pathogenesis.

## 3. Epidemiology

PPID is the most common endocrine disorder of older horses and ponies [26], with the median age at diagnosis being 21 years in a large study that included animals of all ages [27]. The prevalence increases with age [26,28], starting at <3% in animals aged <10 years [29] and increasing up to 20% in horses and ponies aged 15 years and older and 30% in animals over 30 years of age [30]. The youngest animal with histologically confirmed PPID was 7 years old [4]. Whilst several studies suggest that greater proportions of pony breeds are affected by PPPID compared to horses [26,31,32], only one study directly investigated breed as a risk factor for PPID and found no significant difference in the odds of PPID between horses and ponies [30]. The evidence relating to whether sex is a risk factor for PPID is contradictory. Some studies suggest that mares are at an increased risk [4,33], others that males are at an increased risk [32,34] and others that there is no association between sex and PPID [30,35]. Epidemiological data related to PPID in donkeys and mules is lacking; whilst it has been suggested that the prevalence of PPID in donkeys might be higher than in horses and ponies due to their longer life expectancy [36], others report that PPID occurs rarely (1.9% of animals) in donkeys [37,38] and is less common than expected [39].

## 4. Clinical Signs

**Figure 3:**
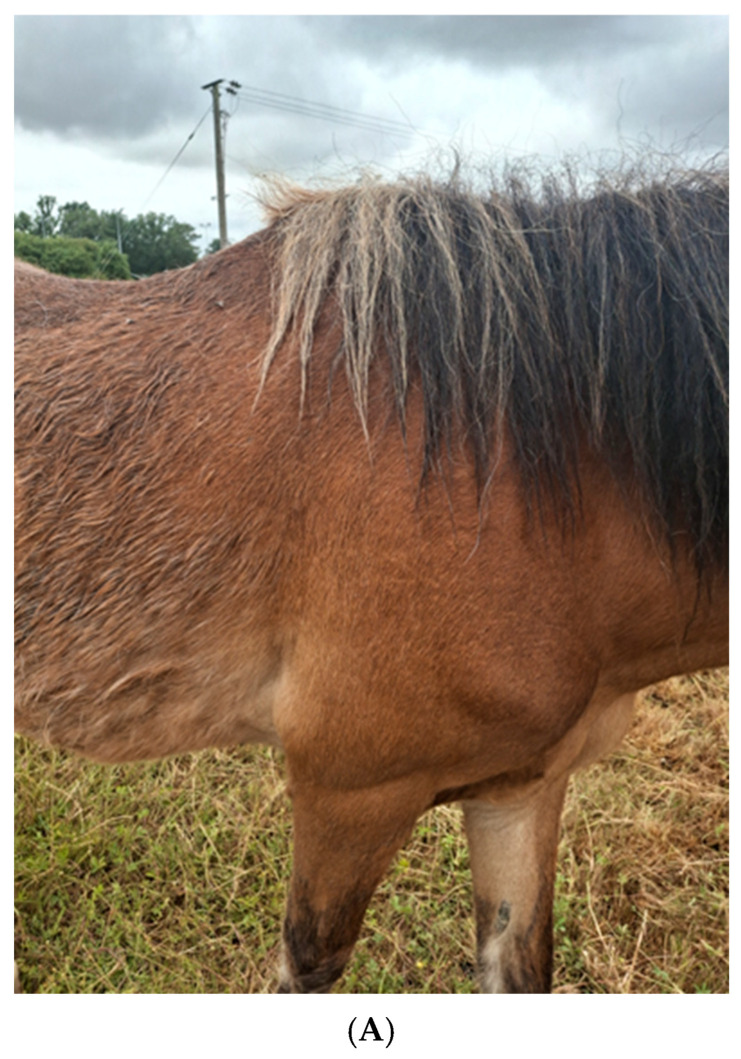

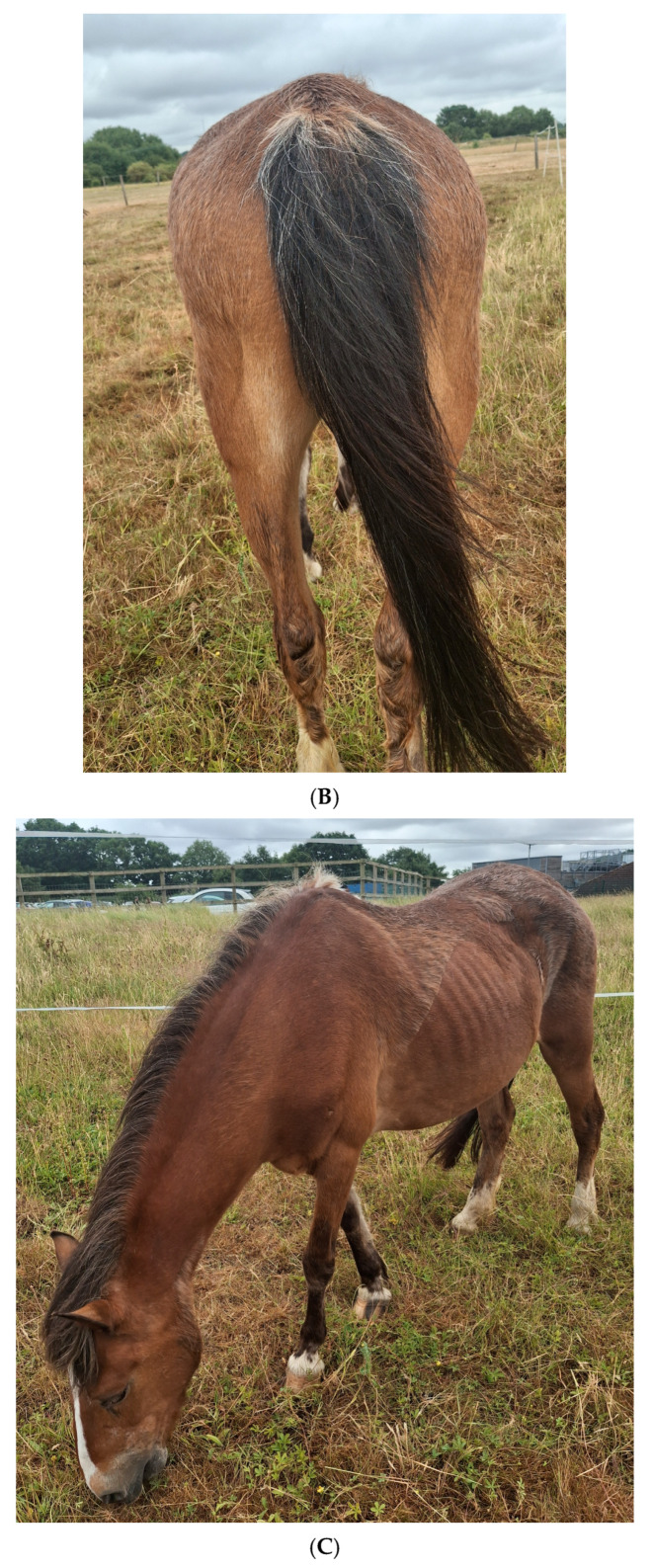
(**A**) Hypertrichosis is the most common clinical sign reported in animals with pituitary pars intermedia dysfunction (PPID); (**B**) epaxial muscle loss provides a moderate level of suspicion of equine pituitary pars intermedia dysfunction (PPID); (**C**) numerous clinical signs provide a moderate level of suspicion that an animal has pituitary pars intermedia dysfunction (PPID), including weight loss.

The clinical signs can be divided into those that are early signs and those that are advanced signs; within each of these sub-divisions, signs can be further divided into those that are strongly suggestive of PPID, those that are moderately suggestive of PPID and those that are possible co-morbidities [6,7]. Regional or generalised hypertrichosis with or without delayed/abnormal haircoat shedding is the most commonly reported clinical sign (Figure 3A) [9,10,29,40,41,42,43,44,45,46,47,48,49,50,51,52], and both hypertrichosis and delayed/abnormal coat shedding reported by the owner are associated with an increased likelihood of an animal having PPID [30,51]. Thus, these haircoat changes are considered strongly suggestive of PPID [6,7]. Numerous clinical signs provide a moderate level of suspicion that an animal has PPID, including hyperhidrosis, abnormal fat distribution/regional adiposity, epaxial muscle atrophy/loss of topline (Figure 3B), laminitis, weight loss (Figure 3C), recurrent infections, behavioural changes/lethargy, polyuria and polydipsia, a pot-bellied appearance, bulging supraorbital fat pads, reduced wound healing, lordosis and infertility [6]. These signs are reported to be identified in animals with PPID with variable frequencies [9,10,29,40,41,42,43,44,45,46,47,48,49,50,51,52], but there is no or limited evidence to suggest that their occurrence is positively associated with an animal having PPID [6]. Many other clinical signs are associated with a low clinical suspicion for PPID, including tachypnoea, anhidrosis, exercise intolerance, polyphagia, ataxia, change in haircoat colour and/or texture, suspensory ligament desmitis and persistent or recurrent endoparasitism [6]. These clinical signs seem to occur at a low frequency in PPID cases reported in the literature and are not positively associated with a diagnosis of PPID [9,10,29,40,41,42,43,44,45,46,47,48,49,50,51,52].

Laminitis is the clinical sign associated with PPID that is perceived to have the potential to have the greatest impact on quality of life. Whilst it was previously thought that all animals with PPID were at an increased risk of developing laminitis, it has become clear that only between 25 and 50% of animals develop this clinical sign [27,53], and laminitis risk is associated with insulin dysregulation (ID). Several studies have demonstrated that animals with PPID and increased circulating insulin concentrations are at a greater risk of developing laminitis compared to animals with PPID and normal insulin concentrations [8,9,10]. Whilst the overproduction of POMC-derived peptides has been speculated to exacerbate ID in cases of PPID through antagonism of insulin [54], any causal association between PPID and ID has yet to be demonstrated, and a cohort study demonstrated that plasma ACTH concentrations and clinical signs suggestive of PPID do not predict future laminitis development risk in currently non-laminitic animals [55]. Thus, the exact relationship between PPID and ID remains to be elucidated.

It should be noted that latitude impacts on the clinical signs seen associated with PPID in horses and ponies. The majority of studies describe the signs seen in animals living in the higher latitudes of the Northern Hemisphere, and description of the disease at lower latitudes and in the Southern Hemisphere is limited [9]. Australian studies have demonstrated that the clinical presentation of PPID changes with latitude and climate, with anhidrosis and polyuria/polydipsia more commonly recognised at lower latitudes [9], and anhidrosis and heat stress with secondary exercise intolerance should also be considered as clinical features of PPID in the hot, humid conditions of a tropical or sub-tropical climate [56].

## 5. Diagnosis

A diagnosis of PPID is made based on the signalment, clinical signs and results of further diagnostic tests. For animals in whom there is a high clinical suspicion of disease based on the clinical signs, i.e., presence of hypertrichosis, delayed or incomplete haircoat shedding, a diagnosis of PPID requires confirmation with further diagnostic testing [6]. For animals in whom there is a moderate clinical suspicion based on the clinical signs, the age of the horse needs to be considered, as well as the presenting signs; thus, in animals younger than 10 years, alternative potential causes of the clinical signs should be considered, due to the rare occurrence of the disease in animals of this age in epidemiological studies [29]. In animals with clinical signs that have a low clinical suspicion of disease, again the age of the animals should be considered alongside the presenting clinical signs and other possible causes investigated in animals less than 15 years old [6].

The two currently recommended diagnostic tests for PPID are measurement of basal plasma ACTH concentrations and measurement of the ACTH response to thyrotropin-releasing hormone (TRH) [7]. Given the fact that ACTH is not the predominant POMC-derived peptide produced by the equine PI, the choice of this hormone as the preferred biomarker rather than any of the other PI products might seem illogical; however, this choice is likely due to ACTH assays being readily available commercially, the lack of availability of assays for other PI-derived peptides outside of the research setting and, potentially, the incorrect historical assumption that PPID was analogous to pituitary-dependent hyperadrenocorticism in other species [57]. Currently no other biomarkers have been found to outperform measurement of basal plasma ACTH concentrations [57], as whilst α-MSH had higher specificity but lower sensitivity than ACTH throughout most of the year and similar diagnostic value in the autumn [30], there is no validated assay currently available within a commercial clinical setting [57]. The dexamethasone suppression test is no longer recommended as a diagnostic test for PPID; thus, if TRH stimulation testing is not feasible, basal ACTH concentration alone should be used for diagnosis, acknowledging that it may be less supportive of a diagnosis in the earlier stages of the disease [7].

Four publications [30,58,59,60] provide data that could be used to calculate the diagnostic test accuracy for basal ACTH concentrations [6], giving overall sensitivity and specificity in the autumn to be 93% and 88%, and in the non-autumn to be 60% and 87%, respectively. The accuracy of the test also varies with season and clinical suspicion and is highest in animals with a high clinical suspicion in autumn (92%) and lowest in these animals in non-autumn (70%) [6]. Similarly, assimilation of the data reported in the one published study that utilised the ACTH response to TRH 30 min after administration [59] gave overall sensitivity and specificity in the autumn of 89% and 98%, and in the non-autumn of 87% and 94%, respectively [6]. The accuracy of the test similarly varies with season and clinical suspicion and is highest in animals with a low clinical suspicion in the autumn (98%) and lowest in animals with a high clinical suspicion in the non-autumn (90%) [6]. Thus, the season and level of clinical suspicion based on age and clinical signs needs to be borne in mind when considering the sensitivity (animals with disease test positive), specificity (animals with disease test negative) and accuracy (ability to differentiate healthy and diseased animals) of both tests.

There are many factors that can impact the diagnostic test results [15,19,59,60,61,62,63,64,65,66,67,68,69,70,71,72,73,74,75,76,77,78,79,80,81,82,83,84] that need to be considered alongside the accuracy of the test itself. Latitude affects basal ACTH concentrations, but other factors also appear to play a role in the differences detected between different geographic locations [18,60,72,73,84] such that the use of geography-specific reference intervals (rather than latitude-specific) might be appropriate [6]. Breed can also impact both basal ACTH concentrations and ACTH concentrations in response to TRH stimulation, especially in the autumn [60,66,67,71,82], such that reference intervals specific to each breed may also be appropriate [6]. Basal ACTH concentrations can increase due to illness [15,19,78], so measurement in critically ill horses to diagnose PPID should be delayed until the illness has resolved [6]. Additionally, basal ACTH concentrations are increased immediately following transport [76,77,85], and in association with acute stress [75,79], mild to moderate laminitis pain [15] and mild to moderate pain from other causes [15]; thus, measurement of basal ACTH concentrations in these circumstances should also be delayed [6]. Diet, feeding and withholding feed all impact basal plasma ACTH concentrations and the ACTH response to TRH stimulation [62,63,66], so repeat sampling of an individual animal (for example, for monitoring purposes) should be carried out under the same dietary conditions [6]. Furthermore, the ACTH response to TRH stimulation should be performed at intervals of at least 24 h and, ideally, at intervals of 2–4 weeks in the non-autumn, whilst short-term variability in test results is much greater in the autumn, limiting diagnostic accuracy during this period [64,65,81].

Tests for insulin dysregulation (ID) should be performed in all animals with PPID to identify the subset of animals that have ID and are, therefore, at an increased risk of developing laminitis [8,9,10]. Equine ID manifests in three ways, namely hyperinsulinaemia, an excessive insulin response to oral carbohydrate and tissue insulin resistance [86], and any combination of these may occur in an individual animal. These manifestations can be identified by measurement of resting (basal) insulin concentrations, performing an oral sugar (OST) or glucose (OGT) test and performing an insulin tolerance test (ITT), respectively [87]. Basal ACTH concentrations do not appear to be negatively impacted by concurrent dynamic tests for ID [62]. However, whilst there was no impact of performing ITT and TRH stimulation tests concurrently [74,80], the TRH stimulation test should not be performed following an OST, due to a negative impact on ACTH stimulation [62]. Thus, the timing of tests for PPID and ID should be carefully considered to avoid any negative impacts of one test on the results of another.

## 6. Management

PPID can be managed but not cured; it is a lifelong condition. Some owners elect to treat the individual clinical signs, e.g., clip the excessive haircoat and provide unrestricted access to water for individuals with polydipsia. Alternatively, pharmacological management can be employed.

The D2 receptor agonist pergolide is licenced/approved for the treatment of equine PPID. Studies have shown that pergolide therapy results in improvement in one or more PPID-associated clinical sign in between 40% and 100% of animals, with the clinical signs improving in the majority of animals (≥76%) in most studies [88]. Reported improvement in specific clinical signs following pergolide treatment includes hypertrichosis in 30–100% of animals, abnormal fat distribution in 0–33%, hyperhidrosis in 15–45%, lethargy/poor performance in 20–47%, muscle wastage in 21–46% and laminitis in 32–75% [32,89,90,91]. Thus, treatment with pergolide results in improvements in the majority of the PPID-associated clinical signs in most animals [6].

Pergolide has also been shown to result in improvements in the laboratory tests, seen as a decrease in basal plasma ACTH concentrations or a return of basal ACTH concentration to within the seasonally adjusted reference range [88]. Return of plasma ACTH concentrations to within reference intervals (not seasonally adjusted) was reported in 58–71% of cases in two studies [32,92], whilst a decrease in plasma ACTH concentrations was reported in 20–55% of cases in four different studies [43,89,93,94]. In a study that used seasonally adjusted reference intervals, basal ACTH concentration normalised in 28% of animals after commencing pergolide therapy and 55% of cases had a ≥75% reduction [93]. In a different study that used both seasonally and geographically adjusted reference intervals, plasma ACTH concentrations returned to normal values in 44% of cases [9]. The ACTH response to TRH stimulation also improved following the initiation of pergolide therapy in 88% of cases and normalised in 24% of cases [95]. In contrast, there was no significant reduction in the basal ACTH concentration in two studies, which compared pergolide-treated animals to non-treated PPID controls [96] or to pre-treatment values [97]. Thus, it would appear that treatment with pergolide results in a decrease in the basal ACTH concentration and improves the ACTH response to TRH stimulation in most cases [6].

Reported side effects associated with pergolide therapy include decreased appetite, diarrhoea, colic, lethargy, weight loss and behavioural changes; these are considered to be rare, apart from anorexia that is reported to occur in 33% of cases [98]. Further side effects such as sweating, dyspnoea and dry mucous membranes have been reported with very high doses of pergolide [99]. Whilst pergolide is associated with harmful cardiac effects in people, there is no evidence that the same in true in horses in the two studies in which this was investigated [100,101]. If side effects occur, therapy should be discontinued until the signs resolve, and then restarted at half of the initial dose before being gradually increased to the target dose [98].

Some horses are refractory to daily oral administration of pergolide, and cabergoline is a dopamine agonist that is available as an extended-release intramuscular injection for horses [102]. A single study reported that weekly injection of extended-release cabergoline (0.005 mg/kg) resulted in a decrease in plasma ACTH concentrations in 60% (13/22) of animals and clinical improvement in 78% of animals [103]. Partial self-limiting inappetence was reported in 30% and lethargy in 17% of treated animals [103].

Historically, the serotonin (5-hydroxytryptamine; 5-HT) antagonist drug cyproheptadine was recommended as an alternative to dopamine agonist therapy [90]. However, there is no evidence that treatment with cyproheptadine, either alone or in combination with pergolide, is superior to pergolide alone [29].

Alternative therapies are often used in animals with PPID, either alone or in combination with pergolide. Use of chasteberry (*Vitex agnus castus*) was reported to result an improvement in the PPID-associated clinical signs based on owner assessment only in two studies [42,44]; however, the improvement in the clinical signs or decrease in basal ACTH concentrations was no different in horses treated with pergolide in combination with chasteberry compared to those receiving pergolide plus a placebo. Additionally, treatment with *Vitex agnus castus* alone was associated with a deterioration of the clinical signs in 13 out of 14 cases, and 9 of these animals were subsequently treated with pergolide in a third study [89]. Thus, whilst chasteberry may or may not result in an improvement in PPID-associated clinical signs, it does not appear to reduce plasma ACTH concentrations and there is a no apparent benefit to combining chasteberry with pergolide therapy [6].

The subset of animals with PPID that have insulin dysregulation (ID) appear to be at an increased risk of developing laminitis and, therefore, a poorer prognosis [8,10]. In most published studies, pergolide therapy did not improve insulin sensitivity [32,96,104,105]; however when pergolide doses were adjusted on a monthly basis according to basal ACTH concentration over a 12-week period, fasting insulin concentrations were reduced [68]. Additionally, in a recent study involving animals with naturally occurring PPID with ID and animals with ID alone, pergolide therapy reduced the insulin response to a standard carbohydrate meal, whilst tissue insulin sensitivity remained unchanged in animals with PPID and ID [106]. Thus, the evidence relating to the effect of pergolide on insulin sensitivity in animals with PPID is conflicting [6] and further research is warranted.

Finally, it is important to monitor the weight and body condition of horses with PPID [7] and any dietary recommendations should be based upon body condition score and evidence of ID. Horses with PPID that are in an ideal body condition and do not have ID should be fed commercially available senior feeds and allowed access to pasture [7], as neither weight gain/loss nor alterations in insulin sensitivity are necessary. In contrast, horses with PPID that are underweight will benefit from an increase in caloric intake via fat supplements and/or alfalfa-based feeds to support weight gain, whilst obese horses with PPID should be fed a lower-calorie diet based on fibre and be encouraged to follow an exercise program if soundness permits [7]. Animals with ID as well as PPID should be fed a low (<10%)-non-structural carbohydrate (NSC) forage-based diet and allowed limited access to pasture [7] to try and improve insulin sensitivity. It should be remembered that the dietary requirements of horses with PPID are likely to change over time and monthly monitoring of weight and/or body condition score by owners is recommended [7], with adjustment of the diet as necessary. Numerous dietary supplements have been suggested for the management of PPID and/or ID, but scientific evidence supporting a beneficial effect is lacking [7].

## 7. Conclusions

Equine PPID is a common disease affecting older equines, associated with neurodegeneration of the inhibitory hypothalamic dopaminergic neurones to the pars intermedia region of the pituitary gland. This neurodegeneration appears to be associated with oxidative damage and abnormal accumulation of the protein α-synuclein, but the initiating cause of this damage remains unclear and further research is required. This loss of inhibitory input results in overproduction of the POMC-derived hormones normally produced by the pars intermedia, as well as hypertrophy, hyperplasia and eventual adenomatous change in the melanotropes. The disease is associated with a wide range of clinical signs, of which regional or generalised hypertrichosis with or without delayed/abnormal haircoat shedding is associated with the highest clinical suspicion. A diagnosis is based on the age, clinical signs and results of further diagnostic tests, and the two currently recommended tests are measurement of basal ACTH concentrations and measurement of the ACTH response to a TRH stimulation test. The sensitivity, specificity and accuracy of these two tests vary according to season and the index of clinical suspicion, and this, as well as the numerous other factors that can impact the test result, should be borne in mind when interpreting the results. Whilst the disease cannot be cured, it can be managed using the dopamine 2 receptor agonist pergolide, and the majority of animals experience and improvements in both their clinical signs and laboratory test results.

## Data Availability

No new data were created or analyzed in this study. Data sharing is not applicable to this article.
